# SHORT-TERM EVALUATION BETWEEN POLYETHYLENE THICKNESS IN PRIMARY TOTAL KNEE ARTHROPLASTY

**DOI:** 10.1590/1413-785220243203e276755

**Published:** 2024-07-22

**Authors:** Rodrigo Sattamini Pires e Albuquerque, Thales Ramos Pizziolo, Octavio Augusto Tome da Silva, Marcelo Alfredo Guerra Monteiro, Sandra Tie Nishibe Minamoto, Alan de Paula Mozella

**Affiliations:** 1. Centro de Cirurgia do Joelho do Instituto Nacional de Traumatologia e Ortopedia, Rio de Janeiro, RJ, Brazil.; 2. Universidade Federal Fluminense, Niterói, RJ, Brazil.

**Keywords:** Knee Arthroplasty, Joelho, Prosthesis Failure, Artroplastia do Joelho, Joelho, Falha de Prótese

## Abstract

**Objective:**

The objective of the research was to carry out a comparative study between Smith & Nephew ® or Zimmer ® prostheses with thick versus thin polyethylene, in patients undergoing primary total knee arthroplasty, during a short-term follow-up. Thus, the objective was to analyze the survival of the implants in question under the clinical and radiographic aspect.

**Methods:**

The sample was divided into two groups: Group 1 with thick polyethylene and group 2 with thin polyethylene. A clinical analysis of the patients was carried out and the implants were checked for loosening.

**Results:**

The groups were similar when compared. According to the Ahlbäck classification, 83% of the patients were in groups IV and V. The median functional score in the postoperative period was similar between the two groups. Postoperatively, the tibiofemoral angle fluctuated between 5 and 6 ^0^ valgus on average. Two complications were observed in each group. None of the evaluated patients presented implant loosening

**Conclusion:**

Patients treated with thick polyethylene had the same functional score as the control group, as well as the absence of radiographic changes in this short-term follow-up, with implant survival and a similar rate of complications between both groups. *Level of evidence III, Retrospective study.*

## RESUMO

**Objetivo:**

O objetivo desta pesquisa foi realizar um estudo comparativo entre as próteses Smith & Nephew ® e Zimmer ®, com polietileno espesso versus o fino, em pacientes submetidos à artroplastia total primária do joelho, durante um seguimento de curto prazo. Dessa forma, foi analisada a sobrevida dos implantes em questão sob o aspecto clínico e radiográfico.

**Métodos:**

A amostra foi dividida em dois grupos: grupo 1 com polietileno espesso e grupo 2 com polietileno fino. Foi realizada análise clínica dos pacientes e verificado se ocorreu soltura dos implantes.

**Resultados:**

Os grupos tiveram resultados semelhantes quando comparados. Segundo a classificação de Ahlbäck, 83% dos pacientes eram dos grupos IV e V. A mediana do escore funcional no pós-operatório foi similar entre os grupos. No pós-operatório o ângulo tíbio-femoral oscilou na média entre 5 e 6 ^0^ de valgo. Foram observadas duas complicações em cada grupo. Nenhum dos pacientes avaliados apresentou soltura do implante.

**Conclusão:**

Os pacientes tratados com o polietileno espesso apresentaram o mesmo escore funcional do grupo controle, assim como ausência de alterações radiográficas nesse seguimento de curto prazo, com sobrevida do implante e índice de complicações similar entre ambos os grupos. Nível de evidência III, Estudo retrospectivo.

## INTRODUCTION

 The longevity of the population and the higher prevalence of patients with osteoarthritis have increased the frequency of indication for total knee arthroplasty (TKA). ^1^ TKA can be defined as a highly complex surgical procedure for the treatment of osteoarthritis, which can have satisfactory and lasting impacts on the improvement of pain, quality of life and patient function, in addition to the correction of deformities and instabilities of origins related to degenerative processes that affect the knee joint. ^2^ TKA presents excellent postoperative outcomes, in relation to implant survival, with rates of more than 95%, in at least 10 years of follow-up. ^3^

 High molecular weight polyethylenes are the most used in TKAs. Their success is due to various properties such as abrasion resistance, impact strength, low coefficient of friction, and to being chemically inert. ^4^

 The factors that affect polyethylene wear in TKA include polyethylene properties, imperfections and thickness, contact area, level, type of stress, coefficient of friction on joint surfaces and prosthesis conformity. ^5^ The fundamental mechanisms of polyethylene wear are adhesion, abrasion and fatigue and, in turn, with increasing stress, a greater amount of debris is produced, causing, in the long term, osteolysis and aseptic loosening of the TKA. ^6^

 Polyethylene thickness relates directly to stress distribution. As a result, the size of this component has become increasingly important. ^7^ Thick polyethylene can show greater wear due to the change in the articular interline. ^8^

 In TKA, polyethylene thickness has a multifactorial character. The size of this component is secondary to factors such as preoperative deformity, bone resections and ligament releases of the knee. ^9^ Therefore, there is no way to predict polyethylene thickness preoperatively. 

 In primary TKA, the literature is scarce and seeks to correlate polyethylene thickness, functional outcome of joint replacement, and aseptic failure rate. ^10^ ^,^ ^11^

The main objective of the research is to conduct a comparative study between Smith & Nephew® or Zimmer® prostheses with thick versus thin polyethylene in patients submitted to primary total knee arthroplasty during a short-term follow-up. Thus, the objective is to analyze the survival of the implants in question from the clinical and radiographic perspective.

## MATERIAL AND METHODS

**Figure 1.**
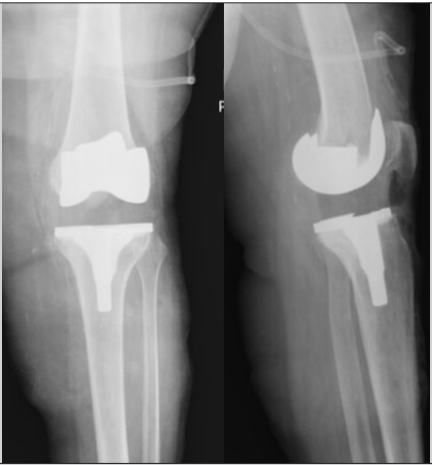
Total knee arthroplasty with thick polyethylene.

**Figure 2.**
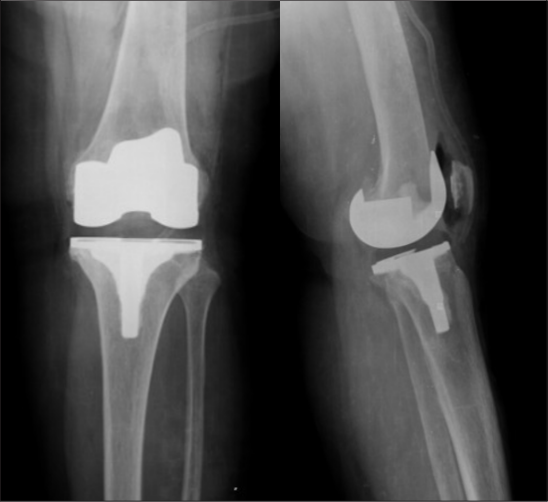
Total knee arthroplasty with thin polyethylene.

 This is an observational, cross-sectional and retrospective study. The participants were identified by using data from the implant sector of our hospital. By identifying patients associated with the specific implant, it was possible to have access to the medical records of those submitted to posterior-stabilized (PS) primary TKA with thick and thin polyethylene. Thus, we conducted a comparative study, observing the radiographic analysis of patients submitted to PS primary TKA, Smith & Nephew® or Zimmer® during a minimum follow-up of 2 years postoperatively. The sample was divided into two groups: group 1 with thick polyethylene (> 14 mm) and group 2 with thin polyethylene (< 14 mm) ^11^ (Figures 1 and 2 ). 

The sample consisted of patients of both sexes and all ages, who had been submitted to primary TKA in the hospital with the Smith & Nephew® and Zimmer® prosthesis type and who had been admitted for treatment during the years 2017 to 2020.

The Smith & Nephew® and Zimmer® prostheses, during this study period, were the implants tendered in our hospital. Thus, the choice of prostheses for analysis is justified, without any type of conflict of interest in the evaluation.

Inclusion criteria: patients submitted to primary arthroplasty performed at the research hospital, with Smith & Nephew® and Zimmer® type prosthesis. Exclusion criteria: failure to document data from the medical record, use of another prosthesis model and non-agreement to participate in the research. The research was approved by the Institutional Ethics Committe (57720022.5.0000.5273) according to the established ethical standards.

 Postoperatively, with a minimum of two years of follow-up, clinical evaluations were performed by a single physician, a member of the Brazilian Society of Knee Surgery and with a graduate degree (doctorate) in Medicine. During the evaluation, patient demographic data were collected, in addition to functional *Knee Society Score (* KSS). ^12^ The KSS form evaluates six variables: pain, function, range of motion, muscle strength, flexion deformity and instability. Subtractions occur in the use of crutches or cane, loss of active knee extension, and instability in varus and valgus. The maximum score is 100 points, of which: 85 points or more is considered excellent; 70 to 84, good; 60 to 69, regular; and 60 or less, unsatisfactory. 

 Radiographic analysis of the PS Smith & Nephew® and Zimmer® implants were performed by another orthopedic physician participating in the study, without prior knowledge of the functional indices obtained during the initial evaluation. The radiographs were performed with bipodal support in the anteroposterior, lateral and axial views of the patella. Radiographic analysis evaluated implant loosening through the criteria used by the *Knee Society Total Knee Arthroplasty Roentgenographic Evaluation and Scoring System* . ^13^ The evaluation of osteolysis consisted in observing the presence of a radiolucent line in the region of the prothesis-cement or bone-cement interface, which was quantified in millimeters of thickness and subsequently analyzed at each radiographic view for comparison. In addition, the degree of osteoarthritis was analyzed by the Ahlbäck classification, ^14^ the type of deformity of the lower limb, as well as the tibio-femoral angle. This angle was calculated by drawing lines between the anatomical axes of the femur and tibia, preoperatively and postoperatively. ^15^ Radiographic data were analyzed using mDicomViewer 3.0 software (Microdata, RJ-Brazil, 2007). 

 Medical records were analyzed by a single physician member of the Brazilian Society of Knee Surgery, and patient demographic data, body mass index (BMI) and *American Society of Anesthesiology (ASA) Classification* were collected. Body mass index was calculated by dividing body mass by height squared. This ratio was recorded in kilograms per square meter (kg/m ^2^ ) as described by Adolphe Quelet. ^16^

Statistical analysis was performed using Microsoft Excel 2016 and GraphPad Prism 5 software. Implant survival was defined as the need for revision for any cause, and survival was determined through the analysis of the Fischer test with a 95% confidence interval. In addition, the Student’s T test of equality of variance was used to calculate the outcomes analyzed with the two independent samples of the population, with a significance level of 0.05.

## RESULTS

A total of 90 patients were evaluated postoperatively after primary total knee arthroplasty, from 2017 to 2020. Patients were divided into two groups: thin polyethylene (49 patients) versus thick polyethylene (41 patients). All patients had been diagnosed with primary osteoarthritis of the knee.

In relation to males, 14 patients with use of thin polyethylene and 11 patients with use of thick polyethylene; in relation to females, 35 patients with use of thin polyethylene and 30 patients with use of thick polyethylene. The distribution by sex was similar between the two groups (p = 0.958).

The mean age of the thin polyethylene group was 70.59 years (standard deviation 7.32) and, of the thick polyethylene group, 67.39 years (standard deviation 7.06). The mean age of the thick polyethylene group was lower than that of the thin polyethylene group (p = 0.038).

We evaluated laterality, right side, there were 25 (56.82%) thin polyethylenes and 19 (43.18%) thick polyethylenes, in relation to the left side, 24 (52.17%) thin polyethylenes and 22 (47.83%). Regarding laterality, the groups were similar to each other (p = 0.818).

In the case of preoperative deformity, it was evaluated and classified according to the Ahlbäck classification, finding with grade II 2 thin and 1 thick polyethylenes, grade III 8 thin and 4 thick polyethylenes, grade IV 18 thin and 10 thick polyethylenes, and grade V 21 thin and 26 thick polyethylenes.

In the thin polyethylene group, BMI was distributed as follows: Normal 6 patients, overweight 19 patients, grade I obesity 18 patients, grade II obesity 5 patients, grade III obesity 1 patient. In the thick polyethylene group, we obtained: Normal 1 patient, overweight 13 patients, grade I obesity 12 patients, grade II obesity 8 patients, grade III obesity 8 patients.

The ASA classification was graded as follows in the thin polyethylene group: Grade I 1 patient, Grade II 44 patients, Grade III 4 patients. In the thick polyethylene group we obtained: Grade I 4 patients, Grade II 33 patients, Grade III 4 patients.

Regarding the preoperative axis, the median of the thick polyethylene group was -8 degrees quartile interval (QI) = (-15.0 – 12.5) and the median of the thin polyethylene group was -5 degrees QI = (-12.0 – 7.0). The groups were similar in relation to the preoperative axis (p = 0.567). The varus axis was considered negative and the valgus axis was considered a positive number.

Regarding the postoperative axis, the median of the thick polyethylene group was 6 degrees QI = (5.0 – 7.0) and the median of the thin polyethylene group was 5 degrees QI = (5.0 – 6.0). The groups were similar in relation to the postoperative axis (p = 0.063). The varus axis was considered negative and the valgus axis was considered a positive number.

Regarding complications, each group presented two complications, totaling four complications. In the thin polyethylene group, one patient presented paresthesia in the operated knee and leg and one patient presented joint stiffness and had to undergo manipulation under anesthesia. In the thick polyethylene group, one patient had infection and one patient had wound dehiscence. None of the patients evaluated had implant loosening.

The mean preoperative objective KSS was similar between the two groups (p = 0.672), and that of the thin polyethylene group was 39.84 (standard deviation = 16.92) and that of the thick polyethylene group was 38.37 (standard deviation = 15.85). The mean preoperative subjective KSS was similar between the two groups (p = 0.253), with the thin polyethylene group being 41.02 (standard deviation = 20.66) and the thick polyethylene group being 35.61 (standard deviation = 23.40).

The median objective KSS in the postoperative period was similar between the two groups (p = 0.938), and that of the thin polyethylene group was 88.00 QI = (84.00 – 92.00) and that of the thick polyethylene group was 88.00 QI = (80.00 – 92.00). The median subjective KSS in the postoperative period was similar between the two groups (p = 0.292), and that of the thin polyethylene group was 82.00 QI = (70.00 – 90.00) and that of the thick polyethylene group was 80.00 QI = (62.50 – 90.00).

## DISCUSSION

There is no study in Brazil that evaluates the thickness of polyethylene and correlates with the functional result of primary TKA, as well as implant survival. In addition, there are few studies in the literature on this topic. We believe that in some developed countries there is no waiting list for surgery; therefore, these patients are operated on at the earliest stage of osteoarthritis and consequently cases have less complexity. As a result, our research becomes extremely relevant.

 Ligament balance, bone resection and polyethylene thickness are interconnected variables. ^7^ As a result, we obtained the preoperative radiographic analysis trying to determine the degree of deformity and correlate with polyethylene thickness. 

 Polyethylene wear can produce debris that influence the loosening of prosthetic components. ^3^ Several variables can influence the frictional wear behavior of polyethylene, such as prosthesis design, raw material used, surgical technique applied, and patient morbidities, such as level of activity and body mass. ^3^ We agree with these statements, therefore, we used two types of prostheses established in the international market. In addition, we assessed BMI. 

 Garceau et al. concluded that there were no differences in TKA revision indices, as well as in the clinical follow-up of thick versus thin polyethylene implants. ^10^ Our study is consistent with this literature; however, these authors analyzed implants with various degrees of constrictions. In addition, they reported that their multicenter study could have generated a lack of standardization. In contrast, Berend et al. observed a higher rate of TKA failure with thick polyethylene. ^9^ Greco et al. also found no difference between the group with thick versus thin polyethylene. ^11^

 Preoperative factors such as degree of deformity, bone loss and ligament insufficiency may affect the choice of polyethylene thickness. ^10^ In addition, this patient profile may present a low functional score. We ratified these statements; however, our research showed a similar postoperative KSS between the two groups. 

 Our study was based on the study of Greco et al., who used the 14 mm polyethylene thickness cutoff limit. Below this limit, it was considered thin, and, above it, thick. ^11^

 Our analysis had a short-term follow-up (2 years) and was based on the study of Greco et al. ^11^

 In the research of Greco et al., the thick polyethylene group was composed of 3.5% of the sample. ^11^ Our study sought to obtain a more homogeneous and proportional population between the two groups (49 thin polyethylenes versus 41 thick polyethylenes). 

 The use of thicker polyethylene was frequent in less experienced surgeons. ^11^ In contrast , our study was composed only of experienced surgeons and members of the Brazilian Society of Knee Surgery, trying to standardize the group. 

Survival analysis of this study demonstrated that “thinner” polyethylenes (< or = 14 mm) had the same short-term performance compared to “thicker” polyethylenes (> 14 mm), assessed on postoperative follow-up imaging and clinically.

 Previously, there were concepts that very “thin” polyethylene in TKAs are associated with higher failure rates, mainly due to wear; however, current studies have shown that “thicker” polyethylenes (≥ 14 mm) are also associated with higher failure rates in medium- to long-term follow-up. ^17^ The surgical variables associated with the implant should be carefully evaluated and may be associated with higher failure rates. ^17^ As a result, we note the controversy on the subject and the relevance of the study. 

In our study, it was observed that most patients were female, but there was no statistical difference between males and females in relation to polyethylene thickness used (thick versus thin); therefore, it is assumed that it is not possible to determine polyethylene thickness in relation to sex.

When evaluating preoperative deformities and the postoperative axis, there was similarity between the groups in our study, with no statistical difference between the groups in relation to polyethylene thickness used.

Our research presents as limitations being retrospective and having a short-term follow-up.

## CONCLUSION

Patients treated with thick polyethylene had the same functional score as the control group, as well as no radiographic changes in this short-term follow-up, with implant survival, complication rate similar between the groups.
